# Competencies for a Healthy Physically Active Lifestyle: Second-Order Analysis and Multidimensional Scaling

**DOI:** 10.3389/fpsyg.2020.558850

**Published:** 2020-12-21

**Authors:** Johannes Carl, Gorden Sudeck, Klaus Pfeifer

**Affiliations:** ^1^Department of Sport Science and Sport, Friedrich-Alexander University Erlangen-Nürnberg, Erlangen, Germany; ^2^Faculty of Economic and Social Sciences, Institute of Sports Science, Eberhard Karls University Tübingen, Tübingen, Germany

**Keywords:** physical activity, health literacy, PAHCO model, physical literacy, validity, physical activity-related health competence

## Abstract

The physical activity-related health competence (PAHCO) model assumes that individuals require movement competence, control competence, and self-regulation competence to lead a healthy, physically active lifestyle. Although previous research has already established some measurement factors (*n* = 8) of the three dimensions, no attempts have so far been made to statistically aggregate them on the sub-competence level. Therefore, the goal of the present study was to test two additional factors for PAHCO and subsequently model the second-order structure with two samples from the fields of rehabilitation and prevention. We conducted two questionnaire surveys with persons with multiple sclerosis (*n* = 475) and teaching students undergoing a basic qualification course in physical education (*n* = 502). After performing exploratory items analysis, we used second-order confirmatory factor analysis (CFA) and multidimensional scaling to investigate whether the scales could be bundled in accordance with the PAHCO model. The CFAs with 10 factors (42 items) demonstrated a good model fit. In contrast, the second-order analysis with a simple loading structure on the three sub-competencies revealed an unacceptable model fit. Instead, a second-order model variant was preferred [comparative fit index (CFI) = 0.926, root mean square error of approximation (RMSEA) = 0.048, standardized root mean square residual (SRMR) = 0.065] in which body awareness and self-efficacy had theory-conform cross-loadings. The results of multidimensional scaling (two-dimensional solution) were in line with the extracted second-order structure. The present results suggested that the extension of the measurement instrument to 10 first-order factors was psychometrically justified for the two populations. The results from the second-order analyses provided the basis for the creation of sum scores, representing manifest indicators of movement competence, control competence, and self-regulation competence. Future studies are needed that cross-validate the extended measurement model with other populations and that relate the sub-competencies of PAHCO to indicators of health-enhancing physical activity.

## Introduction

**Figure 1:**
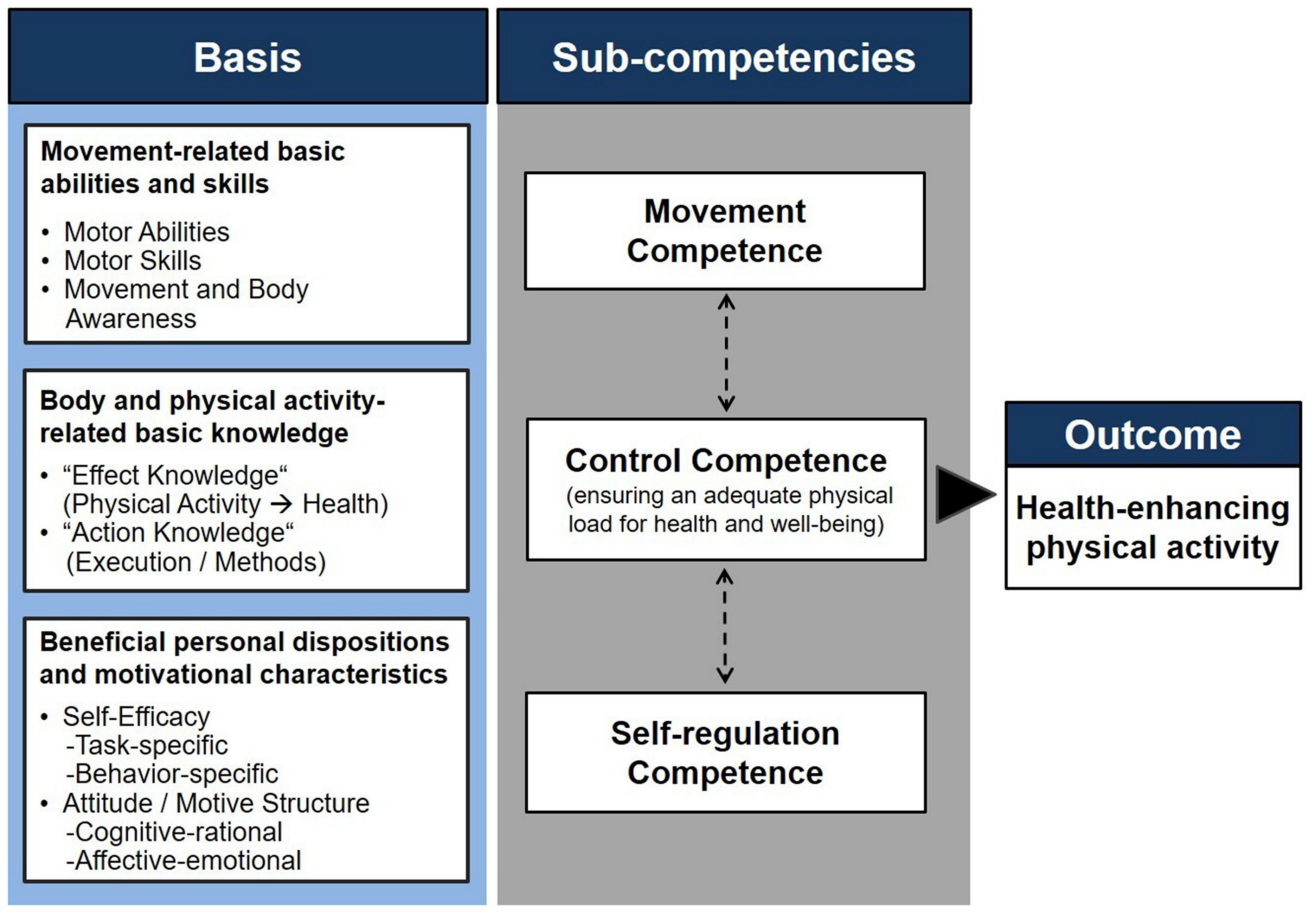
The physical activity-related health competence (PAHCO) model (Sudeck and Pfeifer, 2016).

There is considerable evidence that physical activity (PA) behavior exerts a beneficial effect on individuals' health (Lee et al., 2012; Warburton and Bredin, 2017). Accordingly, having people and populations adhere to a physically active lifestyle can be considered an important goal of our societies. However, several studies have illustrated that a large percentage of individuals is not sufficiently active (e.g., Guthold et al., 2010; Hallal et al., 2012). A large-scale pooling project comprising a total of 1.9 million adults recently revealed that 27.5% of all individuals globally must be characterized as physically inactive, whereby the study has also registered considerable differences between the countries (Guthold et al., 2018). Specific to Europe, the Eurobarometer Study found that 35% of all participants do not exert forms of PA at least once a week (European Union, 2018). In addition, this survey concluded that the percentage of individuals who never do exercise or sport rose from 42 to 46% between the years 2009 and 2017 (European Union, 2018). To counteract such tendencies, the World Health Organization (2018) has released the “Global Action Plan on Physical Activity 2018–2030” (GAPPA) with the recommendation of focusing on individuals' characteristics and behaviors on the one hand as well as on structures and environments on the other. The consideration of these two major pillars is compatible with socioecological theories, pointing out that PA behavior depends on both individual and environmental factors (Bauman et al., 2012; Sallis et al., 2015). With respect to person-related factors, the GAPPA repeatedly suggests addressing people's literacy and competencies (World Health Organization, 2018). Against the background of the frequent use of these two notions, a crucial question arises: What are those competencies and literacy aspects that have to be targeted when people want to adopt or maintain a healthy, physically active lifestyle? When overviewing the literature on health-enhancing physical activity (HEPA) and approaches underlying the two terms “literacy” and “competence,” it becomes apparent that the corresponding descriptions highlight multifaceted and multidimensional requirements for a physically active lifestyle as they integrate physical, motivational, and cognitive aspects (Whitehead, 2007; Sudeck and Pfeifer, 2016; Edwards et al., 2017; Gunnell et al., 2018; Tremblay et al., 2018; Cairney et al., 2019b; Carl et al., 2020b; Martins et al., 2020). In line with this understanding, the literacy and competence approaches assume that an isolated focus on physiological and motor aspects on the one hand (e.g., Lubans et al., 2010) or on motivational and self-regulatory components on the other (e.g., Rhodes et al., 2019) does not account for the complex interplay of personal factors involved when people perform activities on a regular basis. In summary, the approaches using the terms “literacy” and “competence” share a considerable number of commonalities. Nevertheless, there are some conceptual differences (Carl et al., 2020c) that are in line with their separate mentions within the GAPPA (World Health Organization, 2018). The *physical literacy* approach has gained increasing attention during the last two decades (Edwards et al., 2017; Martins et al., 2020) and has put the literacy aspect within the GAPPA on a very solid and elaborate level. However, the approach has not fully exhausted its health potential, since the links to health as an important outcome have not been sufficiently discussed so far (Cairney et al., 2019b). The inclusive character of the framework also comprises competitive and more risky forms of movement (Durden-Myers et al., 2018; Robinson et al., 2018)—forms that may even counteract the promotion of an individual's health. The *competence* concept has the potential to offer a different perspective on physically active lifestyles within the GAPPA by highlighting the domain specificity, context boundedness, goal directedness, and action relatedness of personal factors (Robinson et al., 2015; Carl et al., 2020c). While movement competence as a specific sub-aspect has frequently been the subject of scholarly endeavors (Robinson et al., 2015), there have not yet been academic debates centering on multidimensional competencies for a healthy, physically active lifestyle. To stimulate scientific discussions in this area, the physical activity-related health competence (PAHCO) model has recently been introduced in international literature (Figure 1).

## Theoretical Background and Purpose

The PAHCO model (Sudeck and Pfeifer, 2016) constitutes an integrative framework at the intersection of health literacy and physical literacy (Carl et al., 2020c) that assumes three interrelated and equivalent sub-competencies as essential for a healthy, physically active lifestyle: first, people require *movement competence*, allowing them to participate in planned exercise sessions and be physically active in leisure time (e.g., swimming) or to master important challenges of daily life (e.g., climbing stairs or carrying bags); second, *self-regulation competence* serves as the psychological component, designed to ensure the regular execution of these activities; and third, *control competence* is needed, guaranteeing that individuals not simply apply any stimulus as frequently and intensively as possible. As a rather “qualitative” (Pesce, 2012) domain, this competence component ensures that people do not merely follow the slogan “the more, the better.” Instead, meeting the complex demands of health (Sørensen et al., 2012), this component is geared toward assigning an adequate load to the body for the promotion of physical health and mental well-being. According to the PAHCO model, these three competence areas are, in turn, the result of the integration and coupling of basic elements (see the left side of Figure 1). This assumption harmonizes with theoretical descriptions of competencies in general (e.g., Lichtenberg et al., 2007; Baartman and DeBruijn, 2011). For example, movement competence is formed by the interplay of basic motor abilities, basic motor skills, and a sound body awareness. The basis of control competence is a solid knowledge base in terms of the health-related benefits of PA (effect knowledge) and the appropriateness of certain methods and exercises to achieve these benefits (action knowledge). Finally, the model names positive attitudes toward PA as well as high PA-specific self-efficacy as the basis of self-regulation competence. In addition to these pathways characterizing a transformation of basic elements from the same vertical height, the model also explicitly considers the integration of basic elements from another vertical height into the three competence areas (Sudeck and Pfeifer, 2016; Carl et al., 2020c). For instance, a good body awareness not only contributes to movement competence but can also be used as a feedback source for the identification of an adequate training load (Edwards and Polman, 2013; Smits et al., 2014; Thiel et al., 2018). Another example is the assertion that at least a minimum amount of task-specific self-efficacy is necessary to master given locomotor tasks (movement competence). The PAHCO model has been the subject of some publications in the German-speaking area (Wolters et al., 2016; Gawlik et al., 2018; Hecht, 2020; Schmid et al., 2020) and on the international level (e.g., Sudeck et al., 2018; Carl et al., 2020a; Haible et al., 2020). Also, this framework has already served as the theoretical foundation for interventions and programs (Ley et al., 2014; Streber and Pfeifer, 2018; Bruland et al., 2019; Haible et al., 2019). However, there is currently no diagnostic tool that meets the multidimensional and integrative character of the PAHCO model and, hence, provides consultants, coaches, or therapists with the opportunity to comprehensively assess the competence status of their patients or clients. Nevertheless, two studies including four samples were highly important in the past, as they paved the way for a potential assessment tool (Table 1).

**Table 1:** An overview of the stepwise approach for the assessment development and validation of PAHCO.

**Samples (*n*)**	**Sector**	**Publication**	**Number of included PAHCO aspects**	**Included aspects**
Medical rehabilitation (*n* = 1,028)	Rehabilitation	Sudeck and Pfeifer (2016)	3	Affect regulation, control of physical load, self-control
University sports (*n* = 1,331)	Prevention	Sudeck and Pfeifer (2016)	3	Affect regulation, control of physical load, self-control
COPD rehabilitants (*n* = 351)	Rehabilitation	Carl et al. (2020b)	5	Affect regulation, control of physical load, self-control, emotional attitude, self-efficacy
Apprentices in nursing care and car mechatronics (*n* = 745)	Prevention	Carl et al. (2020b)	8^*^	Affect regulation, control of physical load, self-control, emotional attitude, self-efficacy, MED, MSD, MBD
Persons with multiple sclerosis (*n* = 475)	Rehabilitation	Present study	10	Affect regulation, control of physical load, self-control, emotional attitude, self-efficacy, MED, MSD, MBD, body awareness, cognitive attitude
Teacher students undergoing a basic qualification program in PE (*n* = 502)	Prevention	Present study	10	Affect regulation, control of physical load, self-control, emotional attitude, self-efficacy, MED, MSD, MBD, body awareness, cognitive attitude

*MED, manageability of endurance demands; MSD, manageability of strength demands; MBD, manageability of balance demands; PE, physical education; PAHCO, physical activity-related health competence; COPD, chronic obstructive pulmonary disease*.

* *In exploratory analyses, the ninth factor body awareness has shown bad reliability coefficients. Therefore, it was excluded in this step*.

The goal of these studies was to develop competence-oriented items and multidimensional scales on the sub-competence level. In a first article, Sudeck and Pfeifer (2016) successfully tested three single aspects of PAHCO with two different samples from both the fields of rehabilitation and prevention (Table 1). Inspired by this work, Carl et al. (2020b) recently extended this questionnaire in two consecutive steps, resulting in a five-factor and lastly in an eight-factor measurement model. These measurement models, however, have been developed with two specific samples, which limit the generalizability of the assessment. Therefore, it would be a value *per se* to cross-validate (Blackford, 2017) the measurement models previously extracted. Comparing the current status of the assessment with conceptualizations in publications (Pfeifer et al., 2013; Sudeck and Pfeifer, 2016), two model aspects could still be theoretically considered when striving for a multidimensional operationalization of the sub-competence level. First, no attempts have been undertaken in the context of PAHCO to empirically capture the cognitive-rational attitude component. Second, there is currently no assessment of body awareness. In this context, it is worth mentioning that the exploratory analyses in the second sub-study of Carl et al. (2020b) rejected a first operationalization of the body awareness aspect for both content-related and statistical reasons. Thus, it would be important to reconsider this factor without detaching from a competence-oriented operationalization. Adding the two elements of body awareness and cognitive attitude toward PA to the existing assessment would lead to a 10-factor measurement model. In case of successful testing, it would further be relevant to explore whether the 10 factors can be mathematically pooled into three overarching factors called movement competence, control competence, and self-regulation competence, as theoretically postulated by the PAHCO model. The results of the analysis would be decisive for the creation of sum scores for the three sub-competence areas of the PAHCO model. Such an empirical bundling (Cairney et al., 2019a), in turn, would provide future studies with the opportunity to inspect the associative power of the three sub-competencies (not only of the 10 single scales) with indices of HEPA. Therefore, the goal of the present study was to (1) cross-validate the three-, five-, and eight-factor measurement models on PAHCO with further populations, (2) subsequently investigate the reliability and validity of two further aspects of PAHCO (i.e., body awareness and the cognitive-rational attitude component) including the testing of a 10-factor measurement model, and (3) finally bundle these 10 first-order PAHCO factors to model-conform second-order factors. To achieve these goals, we again used two diverse samples (Table 1) from the two major strands of HEPA, namely, the fields of rehabilitation (Study 1) and prevention (Study 2). The selection of these populations was based on the approval of two research projects in which PAHCO had an important role. Study 1 comprised persons with multiple sclerosis (pwMS). MS is one of the most frequent neurological diseases, for which PA and exercise represent a highly important therapy (Pedersen and Saltin, 2015; Motl and Pilutti, 2016). Study 2 involved teaching students acquiring a basic qualification certificate in physical education. Teachers often report physical complaints and experience considerable mental stress during the workday (Erick and Smith, 2011; von der Embse et al., 2019), which calls for health promotion and, specifically, PA promotion initiatives for future teachers.

## Materials and Methods

### Participants

#### Persons With Multiple Sclerosis (Study 1)

The previous step within the successive validation and assessment development strategy (Table 1) was conducted with a comparably healthy (apprentices) population (Carl et al., 2020b). Since the manageability of balance demands (MBD) factor has not been associated with indicators of PA in the last study (Carl et al., 2020b), we subsequently decided to examine pwMS who typically have problems with motor control (Kister et al., 2013; Rommer et al., 2019). Data were taken from a baseline online survey of the project “MS bewegt” [Engl. *ms moves*], which was specifically installed to launch an Internet-based and competence-oriented program for the promotion of PA in pwMS. Between February and April 2019, voluntary participants were recruited via website newsletters, social network groups, and a mailing list. A total of 484 people followed the link in the message and fully completed the questionnaire survey. We had to exclude the self-report of nine participants due to incomplete consent to data protection (*n* = 6) or not confirming the existence of an official medical diagnosis of MS (*n* = 3). The remaining 475 participants were predominantly female (73.5%), on average 47.8 ± 10.0 years old, and had a body mass index (BMI) of 25.2 ± 5.6 kg/m^2^. The included pwMS had a mean patient-determined disease steps (PDDS) value of 2.74 ± 1.96, with their first official diagnosis being made 15.22 ± 9.27 years ago. Among the participants, 61.9% were undergoing immunotherapy and 62.7% were still employed.

#### Teaching Students (Study 2)

To cross-validate the potential 10-factor measurement model with a different population from the prevention sector, we additionally recruited a sample of teaching students undergoing a basic qualification program in physical education. In Bavaria, Germany, all elementary, middle, and special education teacher candidates must acquire theoretical and practical knowledge in physical education. Depending on individual's educational focus and preferences, physical education can thereby be taken at three different levels, i.e., physical education as a primary, main subject (German: *Hauptfach*), as a secondary, minor subject (German: *Didaktikfach*), or as a third, subsidiary subject (German: *Basisqualifikation*). The basic qualification program is the compulsory course for all elementary, middle, and special education teacher candidates who chose physical education as their third, subsidiary subject (i.e., neither as a primary nor as a secondary subject). Given this preference, it can be assumed that this sample tends to target those individuals who are less interested in or familiar with the topics of PA and health. Within the scope of the PArC-AVE Study (Popp et al., 2020), as a part of the research consortium Capital4Health (here project phase 2), we asked all representatives of the *Working Group Sport Science and Sport of the Universities in Bavaria* (AKS), who coordinate the basic qualification programs in physical education at their universities (*n* = 8), to support the statewide survey in the winter term 2018/2019. All those coordinators were willing to organize the distribution and collection of paper-pencil questionnaires or, if desired, to provide the students with access to an equivalent online survey. This combined assessment strategy led to a final sample of *n* = 502: ongoing elementary school teachers, 61.8%; middle school teachers, 27.0%; and special education teachers, 11.2%. Two coordinators endorsed the organization via online surveys (6.0%, *n* = 30), while six coordinators preferred paper-pencil variants (94.0%, *n* = 472) to increase the response rate. The participants were predominantly female (87.6%), had a mean age of 23.1 ± 3.7 years, and showed an average BMI of 22.6 ± 3.7 kg/m^2^.

### Measures

We used the latest version of the PAHCO questionnaire with eight subscales (Carl et al., 2020b). The *cognitive attitude component toward PA* was measured using a German subscale for the assessment of attitude components in physical exercise (Brand, 2006). This tool comprised four items, rated on a seven-point Likert scale. Within the questions, we replaced the term “sport” with “physical activity” to relate the items to the more inclusive and, for this study, more convenient concept. Since the empirical results from the validation study (Brand, 2006) and our first experiences from a project in the context of pulmonary rehabilitation (Geidl et al., 2017) had shown that participants had some problems with the negatively formulated item “useless,” we decided to modify it by adopting the positively connotated adjective “useful” instead (ATCOG3, see Appendix Table 1). *Body awareness* was assessed with five items on a five-point Likert scale. For the competence-oriented construction of the scale, we followed the basic differentiation between basic sensory and interpretative aspects (Ginzburg et al., 2014) on the one hand (e.g., item BAW2, having a good feeling for one's posture) and more complex aspects of use (e.g., item BAW7, the adequate use of body signals) on the other (Appendix Table 1). We profited from the experiences of a previous study with apprentices in which the initial operationalization of body awareness was not successful (Carl et al., 2020b). Two items were adopted as-is, one item underwent terminological change, and two items were newly developed. All sociodemographic (e.g., age, gender, height, weight), relevant medical (Study 1: e.g., subtype of MS, time since the last relapse), and education data (Study 2: e.g., study program, locality) were captured with self-report questions. However, since there was no validated German self-report tool for the assessment of the severity of the MS disease, we relied on the English version of the PDDS Questionnaire with its nine severity graduations (Learmonth et al., 2013). The first author of this study and a certified German–English translator independently performed a literal translation of this tool, seeking an agreement by consensus afterward.

### Statistics

**Figure 2:**
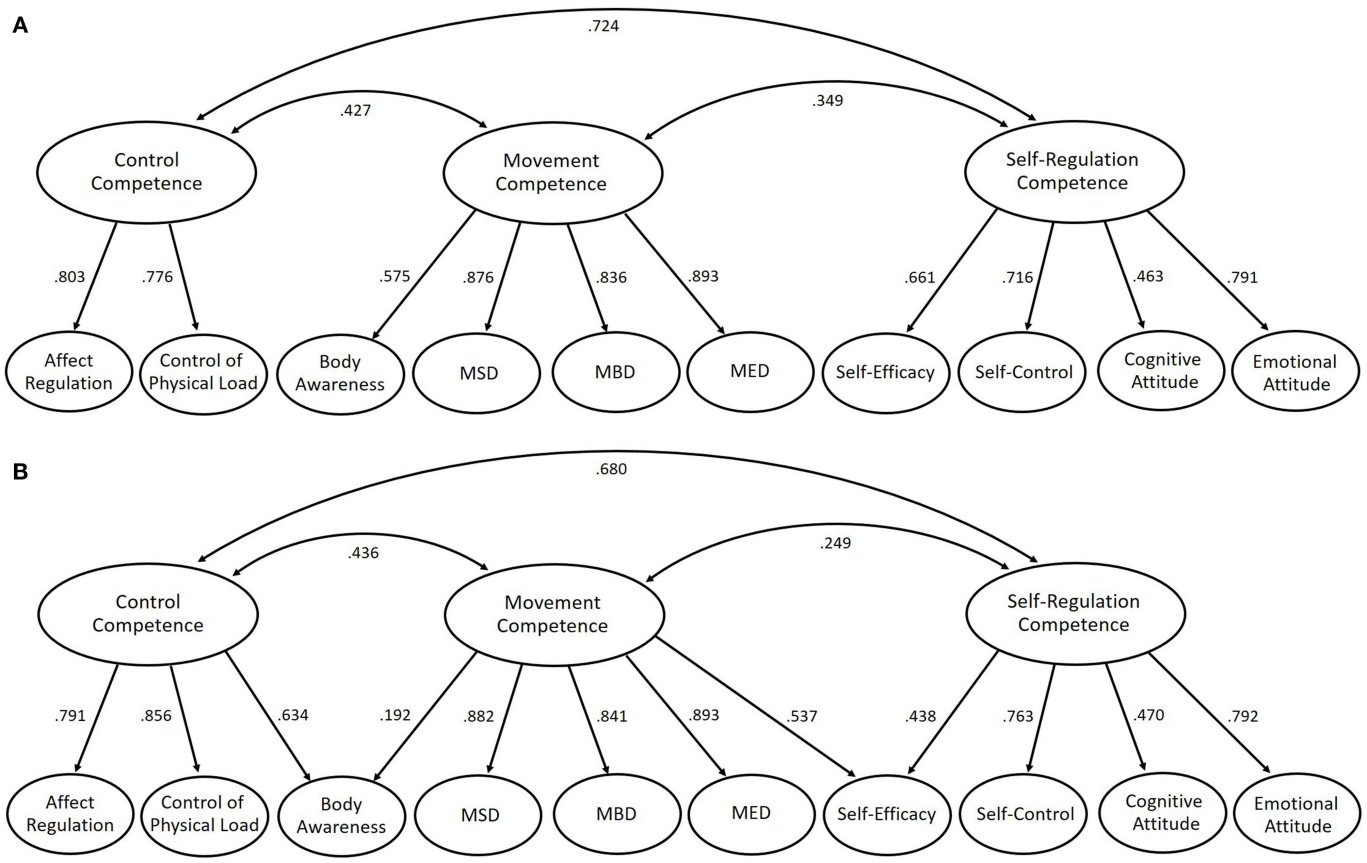
Second-order confirmatory factor analysis (CFA) modeling with the 10 first-order factors **(A)** and a one-on-one allocation (simple structure) **(B)** and two theory-conform cross-loadings. The item level and the correlations between the first-order factors have been omitted in this figure for presentation reasons. The loadings can be retrieved from Tables 2, 3, respectively, whereas the manifest correlations can be taken from Appendix Table 4.

All items were exploratively screened on the basis of common statistical parameters on the one hand (item difficulty, variance within the sample, Cronbach's α, part–whole correlation) and of content-related arguments on the other. As the Mardia test revealed significant violations of multivariate normality (skewness and kurtosis, *p* < 0.001), we relied on robust maximum likelihood estimators (MLR) with Satorra–Bentler scaled statistics to interpret the fit of the models. In addition to the chi-square (SB–χ^2^) statistics, which tend to systematically reject models of high complexity and models that are tested with huge sample sizes (Cheung and Rensvold, 2002), we paid attention to a variant that takes into account the underlying degrees of freedom (SB–χ^2^/*df*). We also followed suggestions by Hu and Bentler (1998), who recommended indicating standardized root mean square residual (SRMR), root mean square error of approximation (RMSEA), and comparative fit index (CFI). To evaluate the magnitude of these coefficients, we adhered to guidelines indicating good (χ^2^/*df* ≤2.0, RMSEA ≤0.05, SRMR ≤0.05, CFI ≥0.95) or satisfactory/acceptable (χ^2^/*df* ≤ 3.0, RMSEA ≤0.08, SRMR ≤0.10, CFI ≥0.90) model fits (Schermelleh-Engel et al., 2003; Weiber and Mühlhaus, 2015). Missing values were treated by applying full information maximum likelihood (FIML) procedures. After the interpretation of the models, we extracted information on indicator and factor reliability. Discriminant validity was determined by following the criterion of Fornell and Larcker (1981), which postulates that discriminant validity is given when the average variance extracted (AVE) of each construct is higher than the squared correlation with any other construct. To inspect whether the 10 factors could be bundled into three overarching factors, we combined both samples (PwMS and teaching students) into one dataset (*n* = 977) and extended the measurement model to a second-level CFA (Chen et al., 2005). More specifically, we computed a 10-factor measurement model with three correlated yet non-overlapping second-order factors (Figure 2A). In accordance with the outlined model assumptions, we statistically pooled manageability of strength demands (MSD), manageability of endurance demands (MED), manageability of balance demands (MBD), and body awareness into a second-order factor called *movement competence*, the factors affect regulation and control of physical load to a second-order factor *control competence* and, ultimately, emotional attitude, cognitive attitude, self-efficacy, and self-control to a second-order factor *self-regulation competence*. Since the model posits that body awareness can be viewed as an important aspect of control competence and self-efficacy an important aspect of movement competence (see *Introduction*), we successively compared this simple loading model (Figure 2A) to a variant that freely estimated these two cross-loadings (Figure 2B). We interpreted the model comparisons by using the Satorra–Bentler scaled chi-square difference test (ΔSB–χ^2^) as well as the information criteria by Akaike (AIC) and Bayes (BIC). Furthermore, we transformed the standardized covariance (correlation) matrix with the first-order factors into a distance matrix (with the formula 1–*r*). Afterward, metric multidimensional scaling (MDS) was performed to more deeply analyze the suitability of a second-order solution and to visually examine the conceptual proximity between the first-order factors. Due to the number of first-order factors (*n* = 10), we limited the analysis to a two-dimensional (*k* = 2) MDS solution (*Q* = 2.25). This decision was supported by the Q coefficient (Backhaus et al., 2015), which would not have surpassed the critical value of 2.00 for three dimensions (*Q* = 1.50). Accordingly, applying more dimensions would have inhibited the interpretability of the solution. All analyses were run with the software R (Version 3.4.3), including the package Lavaan (Rosseel, 2017).

## Results

### Persons With Multiple Sclerosis (Study 1)

There was a good fit for the three-factor [SB–χ^2^/df = 2.14, CFI = 0.978, RMSEA = 0.049 (CI_90_ = 0.039–0.058), SRMR = 0.032] and five-factor [SB-χ^2^/df = 2.18, CFI = 0.973, RMSEA = 0.050 (CI_90_ = 0.044–0.056), SRMR = 0.047] measurement models on PAHCO. The eight-factor variant, which had recently been worked out with apprentices, showed a satisfactory fit [SB–χ^2^/df = 2.82, CFI = 0.938, RMSEA = 0.062 (CI_90_ = 0.058–0.065), SRMR = 0.040], which also demonstrates the validity of the measurement models for this population. Subsequently, we submitted the five new items of the body awareness factor and the four items of the factor cognitive attitude toward PA to exploratory item analysis. Both factors had good internal consistency with Cronbach's α = 0.93. All five items of the body awareness factor ranged in the middle of the scale (item difficulty: 0.58–0.61), thereby displaying no further statistical anomalies. In contrast, there was high agreement to the items of the cognitive attitude toward PA scale (item difficulty: 0.92–0.93), which was associated with high values for skewness and kurtosis (Appendix Table 2). In summary, however, the items did not lie beyond the critical cutoff of 0.95, and, more importantly, there is a content-related argument for this finding. PwMS are typically well aware of the array of beneficial effects resulting from PA, especially when it is executed in a disease-adapted fashion (Frau et al., 2015). Therefore, we continued the development process with the inclusion of these two factors. The CFA with the 10 factors and the 43 items showed a satisfactory model fit [SB–χ^2^/df = 2.48, CFI = 0.931, RMSEA = 0.056 (CI_90_ = 0.053–0.059), SRMR = 0.042]. All items loaded significantly (*p* < 0.001) on their corresponding factor (0.710 ≤ λ ≤ 0.992), and the AVE (0.617–0.906) was good overall (Table 2). Even though the MBD, MSD, and MED factors were highly correlated (0.758 < *r* < 0.834), the Fornell–Larcker criterion was not violated in this sample. The factor reliabilities were consistently located in a good area (0.906 ≤ α ≤ 0.975).

**Table 2:** Analyses of reliability and discriminant validity of the final 10-factor measurement model on physical activity-related health competence (PAHCO) with the sample of persons with multiple sclerosis.

	**Loading**	**Indicator reliability**	**Factor reliability**	**Average variance extracted**	**Highest squared correlation**
**Manageability of Endurance Demands (MED)**			0.941	0.800	0.646
END30	0.891	0.794			
END60	0.930	0.865			
END10s	0.855	0.731			
END30s	0.901	0.812			
**Manageability of Strength Demands (MSD)**			0.929	0.756	0.696
STR15	0.822	0.676			
STR25	0.845	0.714			
STR5m	0.893	0.797			
STR15m	0.914	0.835			
**Manageability of Balance Demands (MBD)**			0.957	0.785	0.696
BAL1	0.873	0.762			
BAL2	0.836	0.699			
BAL3	0.847	0.717			
BAL4	0.940	0.884			
BAL5	0.932	0.869			
BAL6	0.883	0.780			
**Body Awareness**			0.933	0.715	0.572
BAW2	0.783	0.613			
BAW4	0.804	0.646			
BAW3b	0.815	0.664			
BAW7	0.920	0.846			
BAW8	0.897	0.805			
**Control of Physical Load**			0.906	0.617	0.572
CCPL1	0.767	0.588			
CCPL2	0.820	0.672			
CCPL3	0.787	0.619			
CCPL4	0.785	0.616			
CCPL5	0.838	0.702			
CCPL6	0.710	0.504			
**Affect Regulation**			0.947	0.820	0.493
AR1	0.867	0.752			
AR2	0.913	0.834			
AR3	0.945	0.893			
AR4	0.895	0.801			
**Self-Efficacy**			0.918	0.816	0.486
SE1	0.838	0.702			
SE2	0.992	0.984			
SE3	0.873	0.762			
**Self-Control**			0.923	0.801	0.482
SC1	0.869	0.755			
SC2	0.916	0.839			
SC3	0.900	0.810			
**Emotional Attitude**			0.975	0.906	0.493
ATEM1	0.914	0.835			
ATEM2	0.957	0.916			
ATEM3	0.974	0.949			
ATEM4	0.962	0.925			
**Cognitive Attitude**			0.933	0.781	0.260
ATCOG1	0.892	0.796			
ATCOG2	0.856	0.733			
ATCOG3	0.916	0.839			
ATCOG4	0.869	0.755			

**Table 3:** Analyses of reliability and discriminant validity of the final 10-factor measurement model on physical activity-related health competence (PAHCO) with the sample of teaching students.

	**Loading**	**Indicator reliability**	**Factor reliability**	**Average variance extracted**	**Highest squared correlation**
**Manageability of Endurance Demands (MED)**			0.836	0.585	0.246
END30	0.680	0.462			
END60	0.772	0.596			
END10s	0.783	0.613			
END30s	0.818	0.669			
**Manageability of Strength Demands (MSD)**			0.800	0.543	0.171
STR15	0.787	0.619			
STR25	0.803	0.645			
STR5m	0.610	0.372			
STR15m	0.732	0.536			
**Manageability of Balance Demands (MBD)**			0.899	0.594	0.224
BAL1	0.783	0.613			
BAL2	0.756	0.572			
BAL3	0.857	0.734			
BAL5	0.774	0.599			
BAL6	0.672	0.452			
**Body Awareness**			0.841	0.518	0.634
BAW2	0.633	0.401			
BAW4	0.617	0.381			
BAW3b	0.632	0.399			
BAW7	0.863	0.745			
BAW8	0.815	0.664			
**Control of Physical Load**			0.855	0.504	0.634
CCPL1	0.766	0.587			
CCPL2	0.728	0.530			
CCPL3	0.662	0.438			
CCPL4	0.640	0.410			
CCPL5	0.722	0.521			
CCPL6	0.733	0.537			
**Affect Regulation**			0.926	0.763	0.419
AR1	0.821	0.674			
AR2	0.899	0.808			
AR3	0.928	0.861			
AR4	0.841	0.707			
**Self-Efficacy**			0.876	0.738	0.362
SE1	0.759	0.576			
SE2	0.989	0.978			
SE3	0.812	0.659			
**Self-Control**			0.906	0.767	0.372
SC1	0.811	0.658			
SC2	0.920	0.846			
SC3	0.892	0.796			
**Emotional Attitude**			0.920	0.746	0.419
ATEM1	0.823	0.677			
ATEM2	0.876	0.767			
ATEM3	0.894	0.799			
ATEM4	0.860	0.740			
**Cognitive Attitude**			0.871	0.637	0.136
ATCOG1	0.805	0.648			
ATCOG2	0.783	0.613			
ATCOG3	0.792	0.627			
ATCOG4	0.812	0.659			

### Teaching Students (Study 2)

The sample with the teaching students undergoing basic qualification in physical education also revealed good model fits for the three-factor [SB–χ^2^/df = 2.32, CFI = 0.975, RMSEA = 0.052 (CI_90_ = 0.042–0.062), SRMR = 0.034] and five-factor [SB–χ^2^/df = 2.18, CFI = 0.966, RMSEA = 0.049 (CI_90_ = 0.042–0.055), SRMR = 0.042] measurement models. The eight-factor measurement model displayed a satisfactory model fit as well [SB–χ^2^/df = 2.14, CFI = 0.941, RMSEA = 0.048 (CI_90_ = 0.044–0.052), SRMR = 0.047]. The item analysis with the second sample indicated that the fourth item of the MBD factor was too easy for this population, showing an item difficulty of 0.96 and a kurtosis of 19.98 (Appendix Table 3). Following the claim that the assessment must fulfill the basic psychometric requirements in healthy populations as well, we decided to remove this item for all further steps. This decision was supported by the argument that the item covered a comparably easy dynamic locomotor task (maintaining balance while going downstairs). Items 5 and 6 of this factor also referred to stair climbing but included at least a second task (carrying a full shopping bag, carrying a glass full of water), which means that the dynamic locomotor aspect of balance was still sufficiently represented within the remaining item set of MBD when eliminating this particular question. The other items, including those of the two additional body awareness and cognitive attitude toward PA factors, revealed no statistical anomalies. The CFA with the 10 factors and 42 items demonstrated a good model fit [SB–χ^2^/df = 1.98, CFI = 0.933, RMSEA = 0.044 (CI_90_ = 0.041–0.047), SRMR = 0.046]. All items loaded highly significantly on their corresponding factors (*p* < 0.001). Nevertheless, two items showed low regression weights (λ_STR5m_ = 0.61, λ_BAW4_ = 0.62). This finding was tolerated due to the fact that these items had not disclosed any problems in previous studies and that the corresponding indicator reliabilities (0.37 and 0.38) did not fall in an unacceptable area (Weiber and Mühlhaus, 2015). In the sample of teaching students, the AVE was consistently located within an acceptable area (0.504 ≤ AVE ≤ 0.764). However, the AVE of the factors body awareness (AVE = 0.518) and control of physical load (AVE = 0.504) were lower than their squared correlation with each other (*r*^2^ = 0.634). The violation of the Fornell–Larcker criterion indicates that the present assessment could not sufficiently discriminate between these two PAHCO constructs in this sample.

### Investigation of the Second-Order Structure on Physical Activity-Related Health Competence

The stepwise assessment development on PAHCO comprised a total of six different samples (Table 1). Starting with a three-factor variant, the continuous cross-validation and extension strategy led to a 10-factor measurement model. We next examined whether the 10 specified factors could be pooled into three overarching constructs. The simple loading model (Figure 2A) with the merged dataset, however, displayed an insufficient fit [SB–χ^2^/df = 3.63, CFI = 0.913, RMSEA = 0.052 (CI_90_ = 0.050–0.052), SRMR = 0.109], with two statistical indicators lying outside the cutoff values (SB–χ^2^/df > 3.0 and SRMR > 0.10) for acceptable model fits (Schermelleh-Engel et al., 2003; Weiber and Mühlhaus, 2015). Congruent with the assumption of the PAHCO model that body awareness can also be interpreted as an aspect of control competence and self-efficacy as an aspect of movement competence (Pfeifer et al., 2013; Sudeck and Pfeifer, 2016), we further tested a second-order variant in which these two cross-loadings were additionally allowed to be freely estimated (Figure 2B). Even though the SB–χ^2^/df statistics still showed a slightly too high value, the remaining fit indices of this theory-conform second-order CFA were satisfactory [SB–χ^2^/df = 3.24, CFI = 0.926, RMSEA = 0.048 (CI_90_ = 0.046–0.050), SRMR = 0.065], and the SRMR especially turned into an acceptable area. The two cross-loadings, both significant (*p* < 0.001) and substantial in magnitude for a second-order model (λ_BAW−CC_ = 0.634, λ_SE−MC_ = 0.537), contributed to this finding. The pattern revealed that the two loadings even had a stronger conceptual proximity to the second-order cross-factors than to the primary second-order factors (λ_BAW−MC_ = 0.192; λ_SE−SRC_ = 0.438), occupying a larger loading in comparison. In the case of the movement competence to body awareness loading, the reduction was considerable, falling below a value of 0.20. Even though such a decrease is considered significant in some recommendations (Tabachnick and Fidell, 2001), the loading was still highly significant (*p* = 0.004), with the pattern being in accordance with the discriminant validity phenomenon described in the PwMS sample. Nevertheless, the direct comparison between both second-order models (ΔSB–χ^2^ = 317.3, Δdf = 2, *p* < 0.001, ΔAIC = 363, ΔBIC = 358) statistically favored the latter variant of the two solutions. The model results are similar when second-order CFAs are computed for the pwMS and teaching student samples separately (for an overview, see Appendix Figure 1).

**Figure 3:**
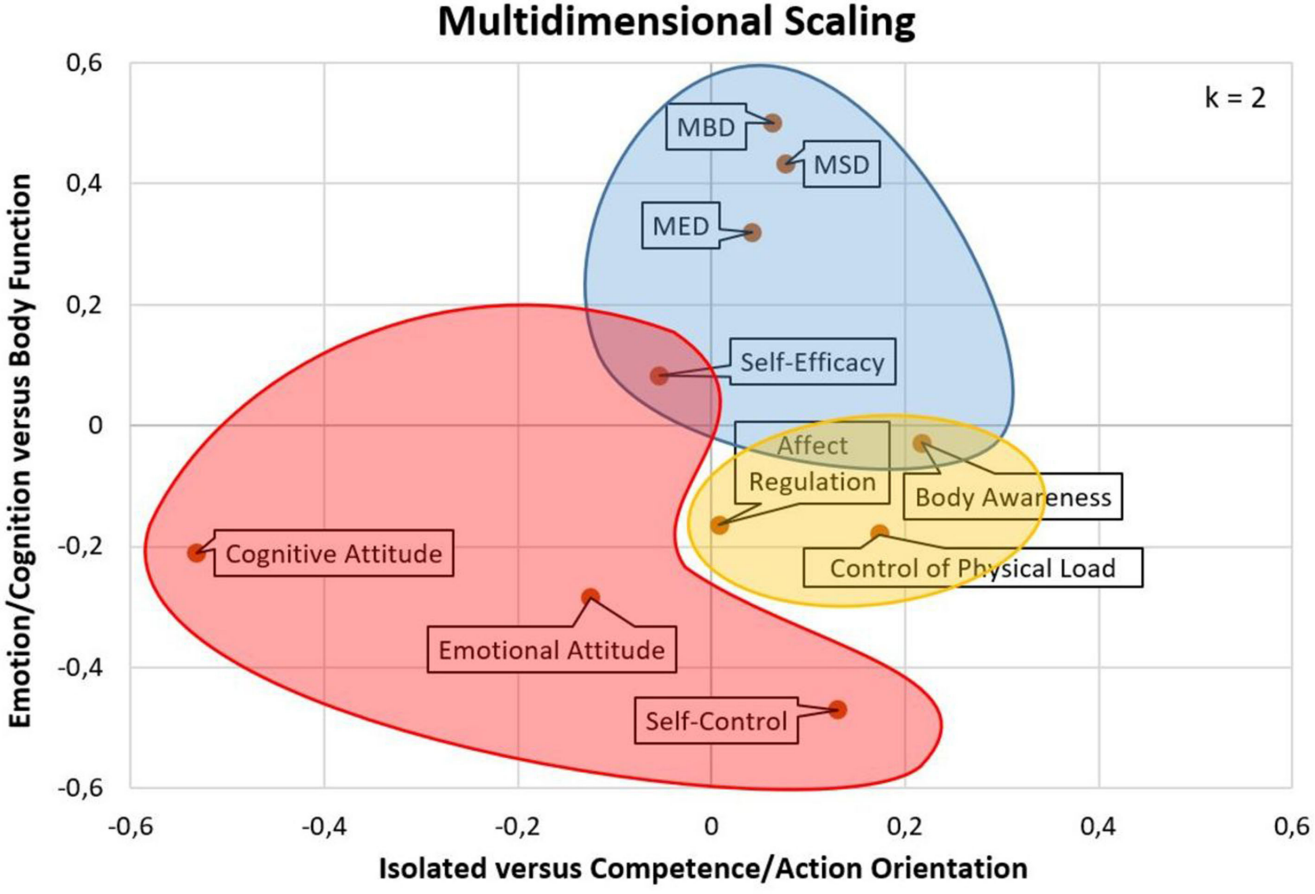
Multidimensional scaling (two-dimension solution).

MDS based on the distance matrix (Appendix Table 4) showed that those factors that could be grouped according to PAHCO could be pooled together spatially. The two cross-factors self-efficacy and body awareness were located at the interface of their corresponding superordinate factors (Figure 3). Movement competence (blue surface) and control competence (yellow surface) occupied a field of limited expansion with a well-interpretable structure. The subdimension of self-regulation competence (red surface) was comparably broad in its conception and operationalization. MDS unfolded that the cognitive attitude component was somewhat outstanding with respect to the other factors, contributing to a graphical expansion of the self-regulation competence surface. Even though the emotional attitude toward PA and the affect regulation factors displayed an empirical proximity, the graphical solution endorsed a theoretical separation between these two PAHCO aspects. After flipping and rotating the 10 points by 95° around the zero of the coordinate system (transformation formula: x' = x · cos α + y · sin α; y' = -x · sin α + y · cos α), the resulting configuration could be interpreted along two dimensions. The x-axis spanned a continuum from a rather isolated level (including the factors of both attitude components) on the left to a complex and more competence-/action-oriented level (such as control of physical load or body awareness) on the right. The points on the y-axis, in contrast, could be ordered from body functions on the top (such as the facets of movement competence MBD, MSD, or MED) to more cognition and emotion-oriented factors (including self-control or the attitude components) at the bottom (Figure 3). In summary, we determined a fit between the configural constellation of the theoretical model (Figure 1) and the empirical data gained through MDS (Figure 3).

Given the model fit of the second-order structure of PAHCO using the basic 10-factor assessment, we were entitled to create sum scores for the 10 first-order factors and the 3 second-order factors in these two specific populations. The two first-order factors body awareness and self-efficacy, which displayed theory-conform cross-loadings (Figure 2B), were included in the score of two competence domains, each with a relative weight of 0.70^1^. Documents on the validated instrument (i.e., the questionnaire instrument, an input mask, an evaluation syntax, and an interpretation guide) can be freely downloaded from a website of the local university^2^ When applying this aggregation procedure, the sum scores of self-regulation and control competence were correlated most strongly across both samples (*r* = 0.709). Albeit slightly lower in magnitude, movement competence was also strongly associated with self-regulation (*r* = 0.496) and control competence (*r* = 0.625).

## Discussion

The present article endorsed the factorial structure of measurement models tested in earlier studies (Sudeck and Pfeifer, 2016; Carl et al., 2020b) using two different samples from the rehabilitation (pwMS) and prevention (teaching students) context. In addition to the eight-factor measurement model, we also included measures of body awareness and cognitive attitude toward PA, thus allowing the investigation of a 10-factor measurement. In summary, the application of the cognitive attitude toward PA scale, transferred and adapted from Brand (2006), was psychometrically warranted. In terms of body awareness, we revised and extended operationalizations that had to be rejected in previous studies (Carl et al., 2020b). The reliability, factor loadings, and overall model fit with the new item set indicated that the operationalization in this study was more adequate. However, although not found in the pwMS sample, the second study with the basic qualification students raised some concerns regarding the discriminant validity of the body awareness factor showing a significant overlap with the control of physical load factor. Indeed, two items (BAW7, BAW8) could also be interpreted as side aspects of the control of physical load factor. Providing a first explanation of the different findings across both samples, sensory inputs could be an integral part of the identification of an adequate physical load among most individuals and pwMS might present a special population who, due to the impaired motor control system, might also have learned to rely on other information sources than their afferent input to regulate their physical load (e.g., feedback from others, intuition, personal experience, guidelines from disease-specific PA recommendations). Second, the ongoing teachers, who have all refrained from choosing physical education as a special subject, could draw on less experience with physical exercise and may therefore prioritize sensory control strategies and more internal foci of attention to arrange strenuous activities (Perkins-Ceccato et al., 2003; Castaneda and Gray, 2007). Third, the possibility cannot be fully excluded that the theoretical construction strategy was not adequately implemented because the items may not capture body awareness as conceptually intended. To accumulate evidence on one of these potential explanations, it is necessary to conduct further studies with other populations. Despite this open point, the present study could extract a theory-conform measurement model that meets the multidimensional and integrative character of the PAHCO model. An initial second-order CFA rejected a first measurement model with a simple loading structure. The free estimation of two cross-loadings significantly improved the model fit. From a theoretical perspective, these two loadings can be directly deduced from two articles introducing the PAHCO model (Pfeifer et al., 2013; Sudeck and Pfeifer, 2016). In line with the statistical fit of the alternative measurement model, it would have been inappropriate to neglect that (a) sound body awareness can contribute to the identification of an adequate physical load in the context of health-oriented exercise (Williams, 2008) and that (b) the execution of motor actions depends on a minimum level of (task-specific) self-efficacy. The subsequent MDS could reproduce the identified second-order structure by mapping the first-order factors along two axes. The graphical representation revealed that the cognitive attitude toward PA factor was slightly outstanding, thus widening the PAHCO and, importantly, the self-regulation surface. Following our interpretation along the x-axis (isolated vs. action-/competence-related orientation), this component may have more characteristics of a basic element, similar to the self-efficacy or the emotional attitude factors, which (as self-regulation elements) also have negative values on the abscissa. In this regard, it would be worth identifying self-regulation elements that are even more competence-oriented. For instance, Sudeck and Pfeifer (2016) suggested taking up the idea of motivational competence that describes an individual's capacity to make motive-congruent decisions (Rheinberg and Engeser, 2010). In this context, it would be crucial to transfer this sub-competence to PAHCO by undertaking a theoretical-conceptual discussion first. From the perspective of behavioral relatedness, technically named criterion validity, it would be necessary to relate the latent second-order factors or sub-competence scores to pivotal outcomes of HEPA (Figure 1). As outcomes, this would, for example, include the volume of PA performed (Sudeck and Pfeifer, 2016; Carl et al., 2020b) or, covering qualitative and health-related aspects of HEPA, parameters such as positive affect (Sudeck et al., 2018), perceived vitality (Schmid et al., 2020), or subjective health (Carl et al., 2020b). Nevertheless, focusing the internal structure of the framework, the present second-order approach could substantiate the integrative and interrelated nature of the PAHCO model. In concert with metatheoretical assumptions, *integrative* means that competencies do not refer to comparably isolated and context-independent (Klieme et al., 2010) movement characteristics such as motor skills. Instead, they require a combination of different abilities and skills, resulting in a multidimensional conceptualization of competencies. Accordingly, to describe a person as competent, the individual should be able to master a number of tasks and demands in different yet concrete situations (Klieme et al., 2010). The present assessment takes this assumption into account using a competence-oriented formulation of items, especially in the area of movement and control competence. The *interrelated* character of PAHCO could be demonstrated by two concrete study results. First, we registered considerable associations *within* the three sub-competencies, i.e., across the different first-order factors. Second, the sum scores *between* the three sub-competencies were correlated, which puts the postulated arrows on the sub-competence level of the PAHCO model (Figure 1) on a stronger empirical basis.

## Limitations

Despite the considerable diversity regarding the examined target groups spanning healthy, young, and well-educated people on the one hand and comparably older individuals with a specific chronic condition on the other, restraint is warranted regarding the external validity of the findings. Currently, it is not possible to generalize the model findings to the population level. More specifically, the selection of the samples was linked to the approval of two research projects, meaning that the previous strategy was so far not able to overcome convenience sampling. In addition, we cannot exclude a selection bias on the project level. In Study 2, the recruitment was realized by contacting the official coordinators of the regional universities. Through their involvement, every Bavarian student of the winter term cohort was personally invited, finally leading to a high participation rate. In Study 1, in contrast, we used different disease-specific social communication channels. This recruitment strategy may have primarily attracted individuals with a special interest in topics of PA and exercise. Furthermore, the present validation was based on different survey formats. While the findings of Study 1 relied on an online format, the insights of Study 2 result primarily from the application of paper-pencil questionnaires (most coordinators preferred this mode to enhance the response rate). Lastly, the PDDS Questionnaire, which provided valuable information for the description of the pwMS sample, has been specifically translated for this study by two independent experts. Future studies should strive for a thorough validation of this instrument.

## Conclusion

The present study built on previous measurement models on PAHCO (Sudeck and Pfeifer, 2016; Carl et al., 2020), cross-validated them, and extended them through the specification of additional operationalizations for body awareness and cognitive attitude toward PA. The 10-factor measurement model showed satisfactory global model fits in pwMS and teaching students. Assuming that the 10 factors have the potential to represent the three sub-competencies of PAHCO in a sufficient (yet not exhaustive) manner, we performed second-order analyses with this set of measurement factors. The second-order confirmatory factor analyses and MDS techniques demonstrated an acceptable model fit for the postulated hierarchical structure when theory-conform cross-loadings for the body awareness and self-efficacy factors are considered. This finding can be interpreted as an empirical rationale for the development of sum scores (movement competence, control competence, self-regulation competence) in these two populations. To the knowledge of the authors, this is the first study that has modeled the sub-competence level of PAHCO in an empirical, multidimensional way. However, further validations are necessary that examine the second-order measurement model in other ideally more representative samples, especially when researchers intend to implement the instrument on a larger scale. Based on the positive findings from the present samples and the previous validation studies, we can specifically recommend the use of the PAHCO instrument (which can be freely accessed on a website) in the fields of rehabilitation and prevention. If researchers plan to implement the instrument with very specific target groups (e.g., if the goal is to promote healthy, physically active lifestyles), it would be valuable to also check aspects of validity.

## Data Availability Statement

Both studies are part of a research project and data will be published as soon as the projects are completed. The data is made available upon request.

## Ethics Statement

The studies involving human participants were reviewed and approved by Landesärztekammer Baden-Württemberg (F-2018-059) and Friedrich-Alexander Universität Erlangen-Nürnberg, Medizinische Fakultät (467_18B). Written informed consent to participate in this study was provided by the participants' legal guardian/next of kin.

## Author Contributions

JC defined the validation strategy, developed the new items, initiated and organized the surveys in both studies, performed the analyses, and drafted the manuscript. GS contributed to the item development and refined the manuscript. KP had the major responsibility for both studies and also refined the manuscript. All authors have approved the final version of the manuscript and agree with the order of presentation of the authors.

## Conflict of Interest

The authors declare that the research was conducted in the absence of any commercial or financial relationships that could be construed as a potential conflict of interest.

## Footnotes

^1^Due to the importance of these two PAHCO aspects resulting from their integrative function, we refrained from using a shared relative weight of 0.50 for each corresponding second-order factor. A relative weight of 1.00, in contrast, would give these two factors too strong an influence among the PAHCO indicators. Therefore, we chose a relative weight of 0.70 for both indictors to feed into the sum score of both corresponding second-order factors.

^2^English Version: https://www.sport.fau.eu/das-institut/forschung/bewegung-und-gesundheit/forschungsprojekte/bgk/ German Version: https://www.sport.fau.de/das-institut/forschung/bewegung-und-gesundheit/forschungsprojekte/bgk/bgk-erstellung-eines-assessmentinstruments/.
