# Supplementary material for: Competencies for a Healthy Physically Active Lifestyle: Second-Order Analysis and Multidimensional Scaling
Source: Front Psychol. 2020 Dec 21;11:558850. doi: 10.3389/fpsyg.2020.558850 (PMC7779792; doi:10.3389/fpsyg.2020.558850)
Supplement: Supplementary file 1 [file Data_Sheet_1.pdf]

Supplementary Material

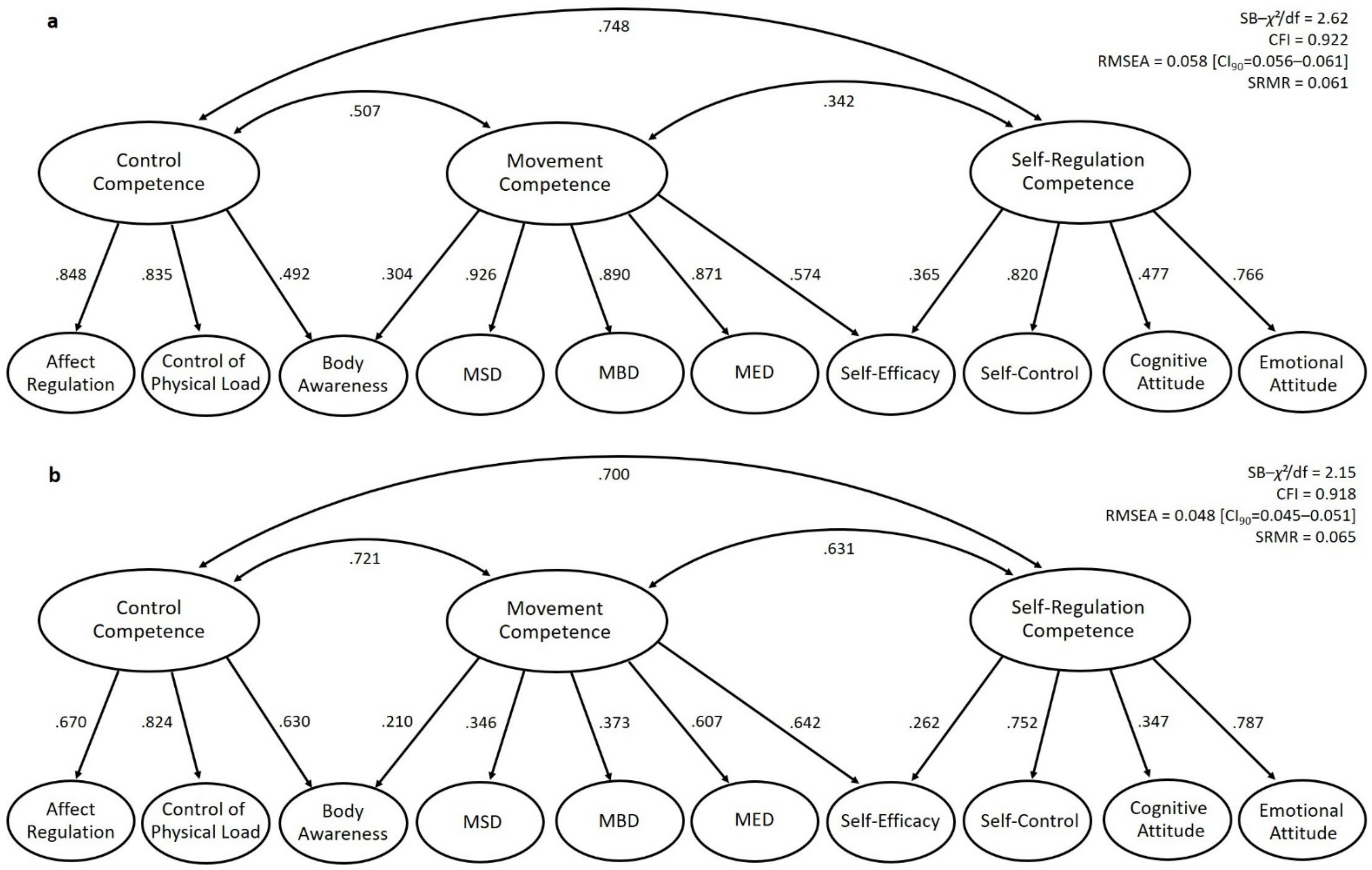

Appendix Figure 1. Sample-specific second-order CFA models
1a) for the multiple sclerosis sample.
1b) for the teaching students (basic qualification in physical education) sample.
Note: The item level and the correlations between the first-order factors have been omitted in this figure for presentation reasons.

Appendix Table 1. *Overview of the different items.*

**Factor**	**Item**	***Item Formulation***
Manageability of Endurance Demands (MED)	END30	I can maintain a slightly exhausting physical activity (e.g., walking, slow running or cycling), which makes me breathe a little faster, for 30 minutes without a break.
	END60	I can maintain a slightly exhausting physical activity (e.g., walking, slow running or cycling), which makes me breathe a little faster, for 60 minutes without a break.
	END10s	I can maintain an exhausting physical activity (e.g., very fast walking, fast running or cycling), which makes me breathe considerably faster, for 10 minutes without a break.
	END30s	I can maintain an exhausting physical activity (e.g., very fast walking, fast running or cycling), which makes me breathe considerably faster, for 30 minutes without a break.
Manageability of Strength Demands (MSD)	STR15	I can lift an object that is approx. 15kg (e.g., full beverage crate, full toolbox).
	STR25	I can lift an object that is approx. 25kg (e.g., full suitcase).
	STR5f	I can lift an object that is approx. 5kg (e.g., full shopping bag, a binder of document files) throughout several floors.
	STR15f	I can lift an object that is approx. 15kg (e.g., full beverage crate, full toolbox) throughout several floors.
Manageability of Balance Demands (MBD)	BAL1	I can stand on one leg and reach for an object without losing balance.
	BAL2	I can stand on tiptoes and reach for an object without losing balance.
	BAL3	I can stand on a chair and reach for an object without losing balance.
	BAL4*	I can walk downstairs without holding on to something without losing balance*.

	BAL5	I can quickly walk downstairs with a full shopping bag without holding on to something without losing balance.
	BAL6	I can carry a glass full of water on a tray going downstairs without spilling water and without losing balance.
Body Awareness	BAW2	I have a good feeling for my posture.
	BAW4	I notice in a timely matter If I physically tense up during a movement.
	BAW3b	My muscles give me timely feedback when I should change my posture.
	BAW7	My body awareness helps me in physically demanding situations.
	BAW8	During strenuous activities I can use signals from my body well.
Control of Physical Load	CCPL1	I am able to adjust my training effort well to my physical condition.
	CCPL2	I know how to use physical training to improve my endurance in the best possible way.
	CCPL3	If my muscles are tensed up, I know exactly how to counter this through physical activity.
	CCPL4	I can use my body signals (pulse, breathing speed) very well to gauge and regulate the amount of physical load.
	CCPL5	If I want to enhance my health by strengthening my trunk muscles (back, stomach), I am confident that I know the right exercises to do.
	CCPL6	I know what to pay attention to in relation to my body in order to avoid excess load or insufficient load.
Affect Regulation	AR1	I am able to regulate my mood through physical activity.
	AR2	If I am feeling down, I can distract myself well through physical activity.
	AR3	I am well able to improve my depressed mood by exercising.
	AR4	I am well able to work off pent-up stress and inner tension through exercise.

Self-Efficacy	SE1	I feel capable to perform physical activities that are challenging for me.
	SE2	I feel capable to perform highly challenging physical activities.
	SE3	I feel capable to perform even the most difficult sport activities.
Self-Control	SC1	If I have planned to exercise, I generally follow through on this plan.
	SC2	I stick with my plan to do exercise and am not easily distracted from that plan.
	SC3	When I decide to do more exercise, I am very disciplined in implementing this plan.
Emotional Attitudes	ATEM1	When I think of being physically active, I feel not/very relaxed.
	ATEM2	When I think of being physically active, I feel not/very content.
	ATEM3	When I think of being physically active, I feel not/very happy.
	ATEM4	When I think of being physically active, I feel not/very comfortable.
Cognitive Attitude	ATCOG1	When I think about it, I believe that being physically active is very healthy.
	ATCOG2	When I think about it, I believe that being physically active is very reasonable.
	ATCOG3	When I think about it, I believe that being physically active is very useful.
	ATCOG4	When I think about it, I believe that being physically active is very worthwhile.

Note: *This item was removed in study two.

Appendix Table 2. *Exploratory item analysis and descriptive statistics in the sample of persons with multiple sclerosis.*

**Study 1 (PwMS)**		*M*	*SD*	P_i	*S*	*K*	r_{PW}	α_{rem}
	END30	3.67	1.46	0.67	-0.70	-0.94	.850	.926

Manageability of Endurance Demands (MED)	END60	3.04	1.62	0.51	-0.05	-1.60	.882	.915
	END10s	3.40	1.57	0.60	-0.40	-1.41	.832	.931
	END30s	2.73	1.57	0.43	0.23	-1.50	.876	.917
Manageability of Strength Demands (MSD)	STR15	3.87	1.40	0.72	-0.88	-0.65	.831	.908
	STR25	3.30	1.50	0.58	-0.30	-1.36	.852	.901
	STR5m	3.51	1.50	0.63	-0.48	-1.25	.833	.907
	STR15m	2.59	1.54	0.40	0.36	-1.42	.820	.912
Manageability of Balance Demands (MBD)	BAL1	3.03	1.51	0.51	-0.04	-1.44	.863	.949
	BAL2	3.08	1.53	0.52	-0.10	-1.48	.840	.951
	BAL3	3.15	1.54	0.54	-0.19	-1.48	.840	.951
	BAL4	3.05	1.61	0.51	-0.07	-1.57	.903	.944
	BAL5	2.72	1.62	0.43	0.26	-1.53	.887	.946
	BAL6	2.71	1.49	0.43	0.23	-1.37	.852	.950
Body Awareness	BAW2	3.39	1.22	0.60	-0.33	-0.81	.745	.928
	BAW4	3.36	1.18	0.59	-0.23	-0.84	.798	.921
	BAW3b	3.30	1.21	0.58	-0.22	-0.83	.807	.920
	BAW7	3.42	1.21	0.61	-0.39	-0.71	.875	.911
	BAW8	3.43	1.22	0.61	-0.42	-0.73	.842	.916
Control of Physical Load	CCPL1	3.68	1.11	0.67	-0.57	-0.31	.715	.892
	CCPL2	3.48	1.28	0.62	-0.46	-0.84	.762	.886
	CCPL3	3.23	1.25	0.56	-0.20	-0.91	.742	.889
	CCPL4	3.41	1.21	0.60	-0.40	-0.80	.741	.889
	CCPL5	3.50	1.25	0.63	-0.50	-0.78	.795	.881
	CCPL6	3.43	1.20	0.61	-0.48	-0.66	.684	.897
Affect Regulation	AR1	3.56	1.10	0.64	-0.41	-0.54	.843	.940
	AR2	3.28	1.35	0.57	-0.24	-1.14	.876	.929
	AR3	3.31	1.24	0.58	-0.26	-0.92	.910	.919
	AR4	3.40	1.34	0.60	-0.36	-1.05	.860	.934
Self-Efficacy	SE1	3.51	1.32	0.63	-0.47	-0.99	.776	.930
	SE2	2.69	1.37	0.42	0.30	-1.14	.931	.802
	SE3	2.10	1.27	0.28	0.93	-0.26	.803	.908

Self-Control	SC1	3.64	1.09	0.66	-0.53	-0.36	.816	.910
	SC2	3.57	1.20	0.64	-0.50	-0.68	.865	.870
	SC3	3.30	1.32	0.58	-0.28	-1.04	.847	.885
Emotional Attitude	ATEM1	5.11	1.70	0.69	-0.81	-0.11	.901	.976
	ATEM2	5.44	1.77	0.74	-1.16	0.38	.940	.965
	ATEM3	5.39	1.76	0.73	-1.13	0.40	.954	.961
	ATEM4	5.37	1.73	0.73	-1.10	0.37	.947	.963
Cognitive Attitude	ATCOG1	6.53	0.91	0.92	-2.77	9.69	.858	.908
	ATCOG2	6.57	0.90	0.93	-3.08	11.88	.819	.920
	ATCOG3	6.57	0.90	0.93	-3.10	12.24	.878	.901
	ATCOG4	6.53	0.99	0.92	-2.96	10.43	.819	.922

Abbreviation: M = Mean; SD = Standard Deviation; P_i = Item Difficulty; S = Skewness; K = Kurtosis; r_{PW} = Part-Whole-corrected Correlation; α_{rem} = Cronbach's Alpha when item removed.

Appendix Table 3. *Exploratory item analysis and descriptive statistics in the sample of teaching students.*

**Study 2 (Teaching Students)**		*M*	*SD*	P_i	*S*	*K*	r_{PW}	α_{rem}
Manageability of Endurance Demands (MED)	END30	4.75	0.57	0.94	-2.28	10.23	.640	.823
	END60	4.19	0.91	0.80	-1.11	0.86	.713	.772
	END10s	4.49	0.78	0.87	-1.67	3.00	.701	.780
	END30s	3.64	1.04	0.66	-0.51	-0.21	.708	.786
Manageability of Strength Demands (MSD)	STR15	4.76	0.56	0.94	-2.83	9.74	.683	.744
	STR25	4.20	0.94	0.80	-1.06	0.60	.690	.715
	STR5m	4.76	0.55	0.94	-2.47	5.85	.560	.786
	STR15m	3.84	0.98	0.71	-0.52	-0.35	.662	.741
Manageability of Balance Demands (MBD)	BAL1	4.45	0.92	0.86	-1.99	3.81	.727	.881
	BAL2	4.52	0.93	0.88	-2.23	4.70	.685	.888
	BAL3	4.74	0.76	0.94	-3.51	12.69	.806	.871
	BAL4*	4.82	0.73	0.96	-4.54	19.98	.797	.873

	BAL5	4.70	0.81	0.93	-3.27	10.98	.770	.875
	BAL6	4.21	0.97	0.80	-1.35	1.70	.630	.899
Body Awareness	BAW2	3.68	1.00	0.67	-0.57	-0.10	.577	.827
	BAW4	3.61	0.94	0.65	-0.43	-0.21	.611	.818
	BAW3b	3.70	0.98	0.68	-0.55	-0.19	.626	.814
	BAW7	3.62	0.97	0.66	-0.32	-0.40	.737	.784
	BAW8	3.64	0.96	0.66	-0.35	-0.46	.678	.800
Control of Physical Load	CCPL1	3.81	0.93	0.70	-0.66	0.23	.664	.828
	CCPL2	3.40	1.10	0.60	-0.24	-0.79	.650	.829
	CCPL3	3.01	1.09	0.50	-0.05	-0.72	.646	.830
	CCPL4	3.46	0.99	0.62	-0.34	-0.31	.610	.839
	CCPL5	3.48	1.20	0.62	-0.48	-0.72	.656	.828
	CCPL6	3.72	0.97	0.68	-0.58	-0.01	.652	.830
Affect Regulation	AR1	3.73	1.06	0.68	-0.66	-0.16	.783	.919
	AR2	3.56	1.25	0.64	-0.52	-0.83	.854	.895
	AR3	3.56	1.20	0.64	-0.52	-0.67	.881	.885
	AR4	3.71	1.19	0.68	-0.72	-0.37	.880	.912
Self-Efficacy	SE1	4.02	1.00	0.76	-1.00	0.58	.684	.891
	SE2	3.29	1.13	0.57	-0.17	-0.75	.884	.706
	SE3	2.49	1.20	0.37	0.33	-0.89	.735	.854
Self-Control	SC1	3.40	1.07	0.60	-0.32	-0.56	.767	.903
	SC2	3.08	1.20	0.52	-0.02	-0.96	.845	.836
	SC3	2.94	1.19	0.49	-0.05	-0.93	.831	.849
Emotional Attitude	ATEM1	4.84	1.38	0.64	-0.53	-0.08	.777	.910
	ATEM2	5.39	1.37	0.73	-0.66	-0.19	.837	.890
	ATEM3	5.15	1.35	0.69	-0.53	-0.13	.843	.888
	ATEM4	5.26	1.32	0.71	-0.56	-0.22	.811	.898
Cognitive Attitude	ATCOG1	6.61	0.68	0.94	-2.35	7.88	.737	.836
	ATCOG2	6.46	0.85	0.91	-2.22	7.16	.714	.839
	ATCOG3	6.46	0.81	0.91	-2.11	6.94	.730	.833
	ATCOG4	6.40	0.91	0.90	-1.83	3.74	.741	.831

Abbreviation: M = Mean; SD = Standard Deviation; P_i = Item Difficulty; S = Skewness; K = Kurtosis; r_{PW} = Part-Whole-corrected Correlation; α_{rem} = Cronbach's Alpha when item removed.
*This item was removed in study two.

Appendix Table 4

Appendix Table 4. *The relationship between the different first-order factors of PAHCO.*

	MED	MSD	MBD	Body Awareness	Control of Physical Load	Affect Regulation	Self-Efficacy	Self-Control	Emotional Attitude	Cognitive Attitude
**MED**	-	0.207	0.270	0.571	0.587	0.526	0.344	0.804	0.635	0.840
**MSD**	0.793	-	0.240	0.583	0.662	0.678	0.449	0.909	0.784	0.911
**MBD**	0.730	0.760	-	0.566	0.707	0.692	0.495	1.000	0.848	0.933
**Body Awareness**	0.429	0.417	0.434	-	0.225	0.516	0.443	0.556	0.596	0.778
**Control of Physical Load**	0.413	0.338	0.293	0.775	-	0.381	0.402	0.402	0.439	0.717
**Affect Regulation**	0.474	0.322	0.308	0.484	0.619	-	0.457	0.413	0.341	0.641
**Self-Efficacy**	0.656	0.551	0.505	0.557	0.598	0.543	-	0.607	0.507	0.607
**Self-Control**	0.196	0.091	0.000	0.444	0.598	0.587	0.393	-	0.414	0.747
**Emotional Attitude**	0.365	0.216	0.152	0.404	0.561	0.659	0.493	0.586	-	0.539
**Cognitive Attitude**	0.160	0.089	0.067	0.222	0.283	0.359	0.393	0.253	0.461	-

Abbreviations: MED = Manageability of Endurance Demands; MSD = Manageability of Strength Demands; MBD = Manageability of Balance Demands.

Note: The standardized covariances (correlations) between the different first-order factors of PAHCO are displayed on the left of the diagonal, the values after the distance transformation (the basis of multidimensional scaling) are displayed on the right side of the diagonal.
